# Chemical control of xylem differentiation by thermospermine, xylemin, and auxin

**DOI:** 10.1038/srep21487

**Published:** 2016-02-16

**Authors:** Kaori Yoshimoto, Hiroyoshi Takamura, Isao Kadota, Hiroyasu Motose, Taku Takahashi

**Affiliations:** 1 Department of Biological Science, Graduate School of Natural Science and Technology, Okayama University, Tsushimanaka 3-1-1, Okayama 700-8530, Japan; 2 Department of Chemistry, Graduate School of Natural Science and Technology, Okayama University, Tsushimanaka 3-1-1, Okayama 700-8530, Japan

## Abstract

The xylem conducts water and minerals from the root to the shoot and provides mechanical strength to the plant body. The vascular precursor cells of the procambium differentiate to form continuous vascular strands, from which xylem and phloem cells are generated in the proper spatiotemporal pattern. Procambium formation and xylem differentiation are directed by auxin. In angiosperms, thermospermine, a structural isomer of spermine, suppresses xylem differentiation by limiting auxin signalling. However, the process of auxin-inducible xylem differentiation has not been fully elucidated and remains difficult to manipulate. Here, we found that an antagonist of spermidine can act as an inhibitor of thermospermine biosynthesis and results in excessive xylem differentiation, which is a phenocopy of a thermospermine-deficient mutant *acaulis5* in *Arabidopsis thaliana*. We named this compound xylemin owing to its xylem-inducing effect. Application of a combination of xylemin and thermospermine to wild-type seedlings negates the effect of xylemin, whereas co-treatment with xylemin and a synthetic proauxin, which undergoes hydrolysis to release active auxin, has a synergistic inductive effect on xylem differentiation. Thus, xylemin may serve as a useful transformative chemical tool not only for the study of thermospermine function in various plant species but also for the control of xylem induction and woody biomass production.

Polyamines such as putrescine and spermidine are small cationic amines present in all organisms that regulate various cellular functions^1^,^2^. In plants, polyamines play a role in physiological processes such as organogenesis, fruit development, stress responses, and cell death; in addition, they serve as precursors of numerous alkaloids^3^,^4^,^5^,^6^,^7^,^8^. The asymmetric tetraamine thermospermine is a structural isomer of the symmetric tetraamine spermine (Fig. 1a) and was first isolated from the thermophilic bacterium *Thermus thermophilus*^9^. Thermospermine is widely distributed throughout the plant kingdom^3^,^10^,^11^, and it has recently been identified as a novel plant growth regulator that represses xylem differentiation and promotes stem elongation in *Arabidopsis thaliana*^12^,^13^. *Arabidopsis acaulis5-1 (acl5-1)* is a loss-of-function mutant of *ACL5*, which encodes thermospermine synthase^12^,^13^, and exhibits excessive xylem differentiation and severe dwarfism^14^,^15^. In contrast, an *Arabidopsis* mutant defective in spermine biosynthesis shows wild-type morphology under normal growth conditions^16^ and spermine has been implicated in responses to biotic and abiotic stresses in plants^3^,^4^,^5^,^6^,^7^,^8^.

In the course of chemical screening, we found that synthetic proauxins remarkably enhance xylem vessel differentiation in *acl5-1* seedlings but not in the wild type^17^,^18^. Proauxin efficiently diffuses into the cells, undergoes cleavage and releases active auxin. The inductive effect of proauxins is completely suppressed by thermospermine, indicating that thermospermine negatively regulates auxin signalling that promotes xylem differentiation. In accordance with this scenario, thermospermine potently suppresses the expression of genes involved in auxin signalling, transport, and synthesis^19^. Together with the fact that *ACL5* expression is up-regulated by auxin^15^ and down-regulated by thermospermine^13^, thermospermine may act in a negative feedback loop against auxin-induced xylem differentiation. A recent study showed that ATHB8, a member of the class III homeodomain-leucine zipper (HD-ZIP III) protein family directly activates *ACL5* expression in this negative feedback loop^20^,^21^. HD-ZIP III transcriptional regulators including ATHB8 redundantly control xylem differentiation and patterning^22^,^23^. However, the molecular mechanism of action of thermospermine remains to be clarified. Polyamine biosynthesis inhibitors have been shown to be useful for the study of polyamine functions^2^. Here, we developed an antagonist of spermidine as an inhibitor of thermospermine biosynthesis and evaluated its inductive effect on xylem differentiation. Our results present new insights into the function of thermospermine in plants and on the chemical manipulation of xylem differentiation.

## Results

### Xylemin promotes xylem differentiation by suppressing thermospermine biosynthesis

**Figure 1:**
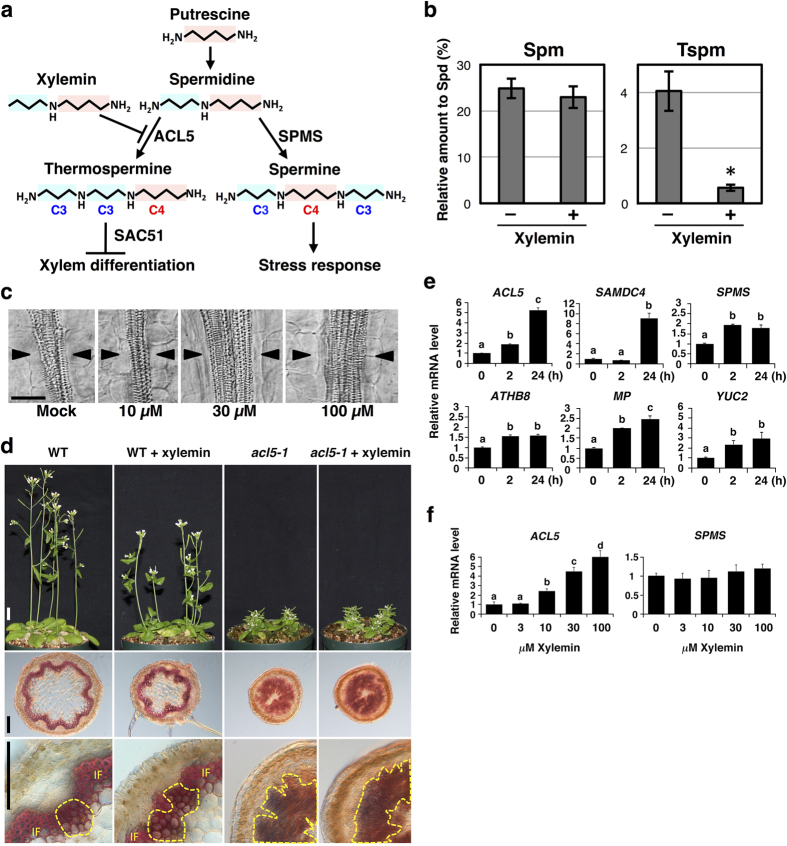
Xylemin promotes xylem differentiation by the inhibition of thermospermine synthesis. (**a**) Biosynthetic pathway of polyamines in plants. (**b**) Effects of xylemin on the levels of spermine (Spm) and thermospermine (Tspm). The 7-day-old wild-type seedlings of *Arabidopsis* were transferred to the liquid medium with 10 μM spermidine (-Xylemin) or with 10 μM spermidine plus 30 μM xylemin (+Xylemin) and grown for 24 hours. The levels of polyamines were analyzed by HPLC. Data are displayed as averages ± SD (*n* = 3). Asterisk indicates significant differences from the value in the mock treatment (Student *t*-test, *P* < 0.01). (**c**) Effect of various concentrations of xylemin on xylem differentiation in hypocotyls. The wild type seedlings were germinated and grown for 7 days in the liquid medium with different concentrations of xylemin. Scale bar: 50 μm. (**d**) Shoot morphology and xylem differentiation in inflorescence stems of wild-type (WT) and *acl5-1* plants treated with or without xylemin. Xylemin solution at the concentration of 100 μM was daily applied to each shoot apex. Xylem is indicated as the area enclosed by yellow dashed line. IF: interfascicular fiber. Scale bars: 1 cm (upper panels); 100 μm (middle and lower panels). (**e**) Response of gene expression to xylemin. Total RNA was isolated from the wild type seedlings grown for 7 days in the liquid medium and treated for 2 or 24 hours in the medium supplemented with 50 μM xylemin. All transcript levels are relative to those in mock-treated seedlings. Data are displayed as averages ± SD (*n* = 3). Different letters indicate Tukey’s HSD groupings (*P* < 0.05). (**f**) Effect of various concentrations of xylemin on expression of *ACL5* and *SPMS*. Total RNA was isolated from the wild type seedlings grown for 7 days in the liquid medium with different concentrations of xylemin. All transcript levels are relative to mock controls. Data are displayed as averages ± SD (*n* = 3). Different letters indicate Tukey’s HSD groupings (*P* < 0.05).

Thermospermine synthase ACL5 catalyses the transfer of the aminopropyl group from decarboxylated *S*-adenosylmethionine to the aminopropyl side of spermidine (Fig. 1a). Therefore, we hypothesized that *N*-propyl-1,4-diaminobutane, namely, *N*-propylated putrescine, is a potential inhibitor of ACL5 because of the loss of amino group, which is required for the transfer of the aminopropyl group in thermospermine biosynthesis. We designed a synthetic scheme and successfully synthesized this compound by Ns-strategy^24^, in which 2-nitrobenzenesulfonamide (Ns) is used for both protecting and activating the group (see Methods). We named the compound as xylemin after its xylem-inducing activity, which is described below in detail.

To examine the effect of xylemin on the biosynthesis of thermospermine, wild-type seedlings were incubated for 24 hours with 30 μM xylemin and 10 μM spermidine. The level of thermospermine was drastically reduced in these seedlings compared with that of the seedlings incubated only with spermidine (Fig. 1b). However, the level of spermine was not significantly reduced in the seedlings treated simultaneously with xylemin and spermidine (Fig. 1b). Furthermore, while wild-type seedlings treated for 24 hours with 30 μM xylemin showed no reduction in the endogenous levels of both spermine and thermospermine, those grown for 10 days with xylemin showed significantly reduced levels of thermospermine (Supplementary Fig. S1).

Next, we examined the effect of xylemin on xylem differentiation. When grown with different concentrations of xylemin, wild-type seedlings showed obvious enhancement of xylem vessel differentiation in the hypocotyl at concentrations greater than 30 μM (Fig. 1c). We detected the enhancement of xylem differentiation only 12 hours after application of xylemin (Supplementary Fig. S2). Furthermore, daily application of xylemin to the shoot apex of wild-type seedlings remarkably repressed stem growth while resulting in excess xylem differentiation in stem internodes (Fig. 1d). Xylemin did not affect stem growth and xylem differentiation in *acl5-1* (Fig. 1d).

To examine the effect of xylemin on gene expression, 7-day-old seedlings were treated with xylemin for 2 or 24 hours. Transcript levels of *ACL5* and *SAMDC4* were strongly increased by xylemin (Fig. 1e). *SAMDC4* encodes an *S*-adenosylmethionine decarboxylase that donates an aminopropyl group specifically for thermospermine synthesis and its expression is up-regulated in *acl5-1* and down-regulated by exogenous thermospermine^13^,^19^. Expression of spermine synthase (*SPMS*) was slightly increased by xylemin treatment (Fig. 1e). We also examined expression of the genes involved in the regulation of vascular differentiation. ATHB8 is known to function redundantly with other HD-ZIP III members in vascular development^22^,^23^. MONOPTEROS (MP) is an auxin-responsive transcription factor (ARF5) that mediates auxin-induced procambium formation^25^,^26^. MP directly induces ATHB8 expression at preprocambial stages^23^. Transcript levels of these genes are increased in *acl5-1*^19^. These genes were also up-regulated by xylemin (Fig. 1e). In addition, expression of an auxin biosynthetic gene *YUCCA2* (*YUC2*) was also increased by xylemin treatment. When wild-type seedlings were grown for 7 days with different concentrations of xylemin, the transcript level of *ACL5* was increased with 10 μM or more xylemin, but transcription levels of *SPMS* were not altered (Fig. 1f). These results indicate that xylemin affects the feedback regulation of polyamine biosynthetic genes and promotes expression of key regulators involved in vascular development.

### Thermospermine suppresses the effect of xylemin on xylem differentiation

**Figure 2:**
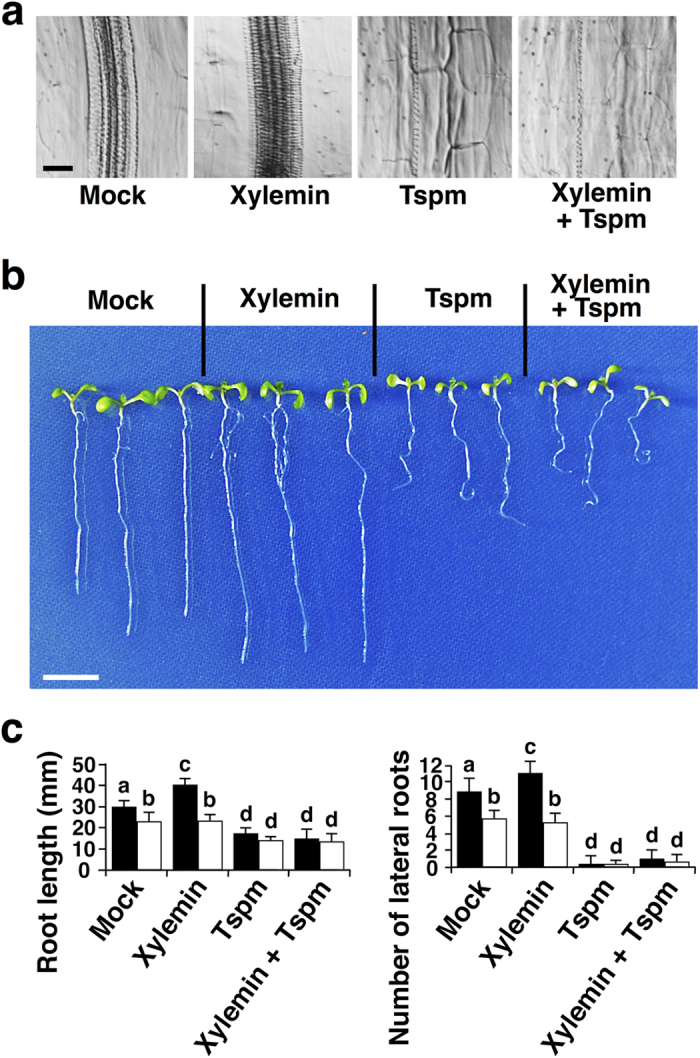
Thermospermine suppresses effect of xylemin on xylem differentiation and root growth. (**a**) Effect of xylemin and thermospermine (Tspm) on xylem differentiation in hypocotyls. The wild-type seedlings were germinated and grown for 7 days in the liquid medium without (Mock) or with 50 μM xylemin and/or 100 μM thermospermine (Tspm). Scale bar: 20 μm. (**b**) Effect of xylemin and thermospermine (Tspm) on seedling development. The wild-type seedlings were grown as in (**a**). Scale bar: 1 cm. (**c**) Quantification of main root growth (left panel) and lateral root formation (right panel). Black and white bars indicate wild type and *acl5-1*, respectively. Data are displayed as averages ± SD (*n* = 10 plants). Different letters indicate significantly different values from each other (Student *t*-test, *P* < 0.01).

To determine whether the effect of xylemin is negated by thermospermine, thermospermine was simultaneously added with xylemin to the liquid medium. Exogenous addition of thermospermine significantly suppressed xylemin-inducible xylem differentiation (Fig. 2a). We also examined the effect of xylemin and thermospermine on root growth. Xylemin promoted main root growth and lateral root formation in the wild type but not in *acl5-1*, whereas thermospermine suppressed them in both seedlings (Fig. 2b,c).

### A proauxin remarkably enhances the inductive effect of xylemin on xylem differentiation

**Figure 3:**
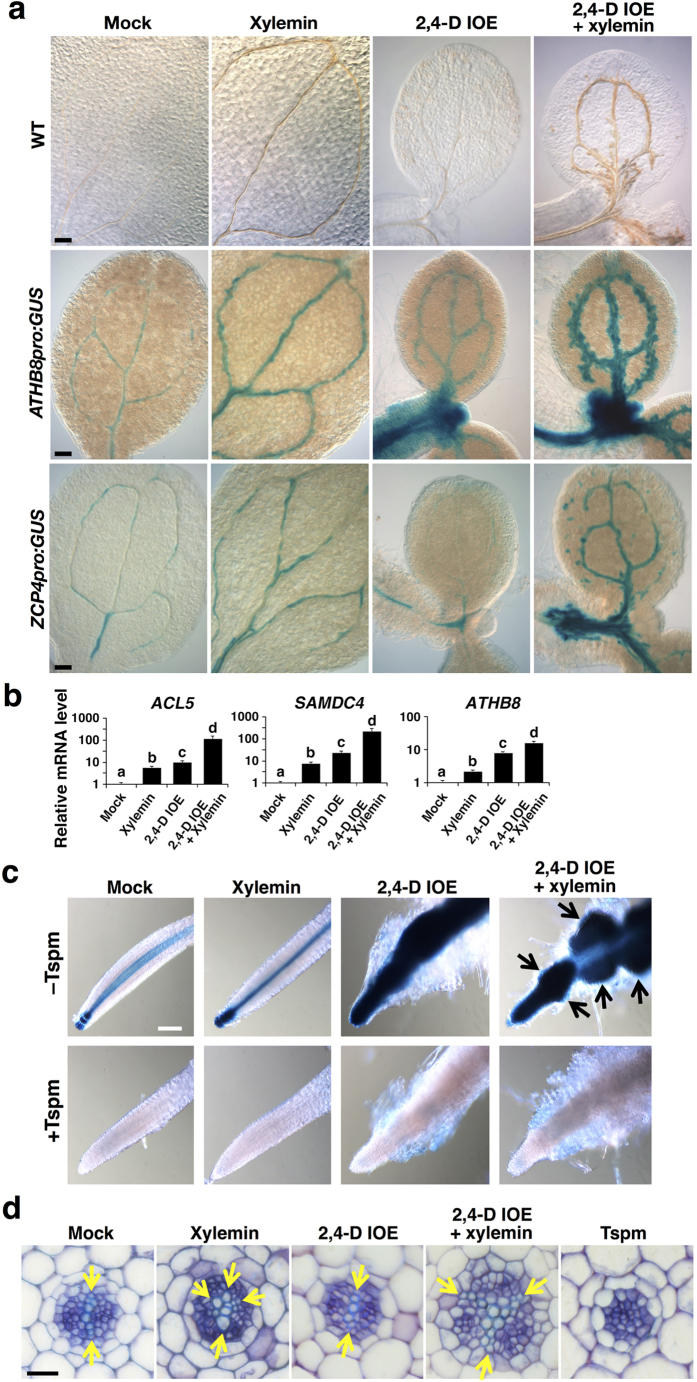
Auxin enhances inductive effect of xylemin on xylem differentiation. (**a**) Effect of xylemin and 2,4-D IOE (auxin prodrug) on xylem differentiation in the wild type (upper panels) and on expression pattern of *ATHB8pro:GUS* (middle panels) and *ZCP4pro:GUS* (lower panels). The wild-type, *ATHB8pro:GUS*, or *ZCP4pro:GUS* seedlings were germinated and grown for 7 days in the liquid medium without (Mock) or with 50 μM xylemin and/or 3 μM 2,4-D IOE. Scale bars: 100 μm. (**b**) Effect of xylemin and 2,4-D IOE on gene expression. Total RNA was isolated from the wild type seedlings germinated and grown for 7 days in the liquid medium without (Mock) or with 50 μM xylemin and/or 3 μM 2,4-D IOE. All transcript levels are relative to those in mock-treated seedlings. Data are displayed as averages ± SD (*n* = 3). Different letters indicate Tukey’s HSD groupings (*P* < 0.05). (**c**) Effect of xylemin, thermospermine (Tspm), and 2,4-D IOE on expression of *ATHB8pro:GUS* in roots. The *ATHB8pro:GUS* seedlings were germinated and grown for 7 days in the liquid medium without (Mock) or with 50 μM xylemin, 3 μM 2,4-D IOE, and/or 100 μM thermospermine (Tspm). Arrows indicate induction and fasciation of lateral roots. Scale bar: 100 μm. (**d**) Effect of xylemin, thermospermine (Tspm), and 2,4-D IOE on xylem differentiation in roots. The wild type seedlings were germinated and grown for 7 days in the liquid medium without (Mock) or with 50 μM xylemin (Xylemin), 3 μM 2,4-D IOE (2,4-D IOE), 50 μM xylemin plus 3 μM 2,4-D IOE (Xylemin +2,4-D IOE), or 100 μM thermospermine (Tspm). Arrows indicate xylem vessel elements. Scale bar: 20 μm.

We next investigated whether or not the synthetic auxin prodrug 2,4-dichlorophenoxyacetic acid isooctyl ester (2,4-D IOE) enhances xylem differentiation in xylemin-treated plants similarly to the *acl5-1* mutant^17^,^18^. Xylemin promoted xylem vessel differentiation in wild-type cotyledons, whereas 2,4-D IOE alone did not, although 2,4-D IOE treatment affected cotyledon expansion (Fig. 3a). The growth of wild-type seedlings in the presence of both 2,4-D IOE and xylemin strongly enhanced the effect of xylemin on xylem vessel differentiation (Fig. 3a). The effect of 2,4-D IOE was also confirmed by the expression of the *GUS* reporter gene fused to the *ATHB8* promoter (*ATHB8pro:GUS*). Xylemin treatment enhanced *ATHB8pro:GUS* expression in veins (Fig. 3a). Because *ATHB8* expression is auxin-responsive, 2,4-D IOE solely induced *ATHB8pro:GUS* expression, especially in hypocotyls. Simultaneous addition of xylemin and 2,4-D IOE synergistically enhanced *ATHB8pro:GUS* expression around veins (Fig. 3a). The synergistic effect of xylemin and 2,4-D IOE was also observed by the expression of a xylem vessel-specific marker, *Zinnia Cysteine Protease 4* (*ZCP4pro:GUS*), which was not up-regulated solely by 2,4-D IOE (Fig. 3a). We further examined the effect of 2,4-D IOE and xylemin on gene expression and found that the transcript levels of *ACL5*, *SAMDC4*, and *ATHB8* were increased by about 100-fold by the co-treatment with xylemin and 2,4-D IOE (Fig. 3b).

The effects of xylemin, thermospermine, and 2,4-D IOE were also examined in root tissue. Xylemin promoted xylem differentiation in the root, whereas thermospermine almost completely blocked it (Fig. 3c,d). Excess xylem formation in xylemin-treated wild-type roots was reminiscent of that of untreated *acl5-1* (Supplementary Fig. S3). Xylemin and 2,4-D IOE synergistically promoted xylem formation (Fig. 3c,d). The roots treated with xylemin showed stronger *ATHB8pro:GUS* expression compared to that untreated, whereas the roots treated with 2,4-D IOE showed much stronger GUS staining associated with callus formation (Fig. 3c). Co-treatment with 2,4-D IOE and xylemin resulted in the disorder of cell files, probably indicating enhanced induction and fasciation of lateral roots (Fig. 3c). When thermospermine was added simultaneously with xylemin and/or 2,4-D IOE, neither GUS staining nor the disorder of cell files was observed (Fig. 3c). These results appear to be consistent with the contrasting functions of thermospermine and auxin in xylem differentiation and lateral root formation, and that xylemin is an inhibitor of thermospermine biosynthesis.

Our previous study has identified suppressor mutants of *acl5-1*, named *sac*, which recover the dwarf phenotype in the absence of thermospermine. *SAC51* encodes a basic helix-loop-helix (bHLH) protein whose translation is enhanced by thermospermine and may play a key role in thermospermine-dependent suppression of xylem differentiation^27^,^28^. In the *sac51-d* dominant allele, *SAC51* translation may occur independent of the control by thermospermine. Thus, we examined the effect of xylemin in *sac51-d* and confirmed that xylem differentiation was not enhanced by xylemin in *sac51-d* (Supplementary Fig. S4). In addition, xylemin did not affect xylem differentiation in *acl5-1* (Supplementary Fig. S4). In contrast to the wild type, *sac51-d* was also not responsive to xylemin in the transcript levels of *ACL5*, *SAMDC4*, and *ATHB8* (Supplementary Fig. S5). These results suggest that the effect of xylemin on xylem differentiation is mediated by its effect on thermospermine-dependent expression of the SAC51 function.

### Xylemin induces xylem differentiation in other plants

**Figure 4:**
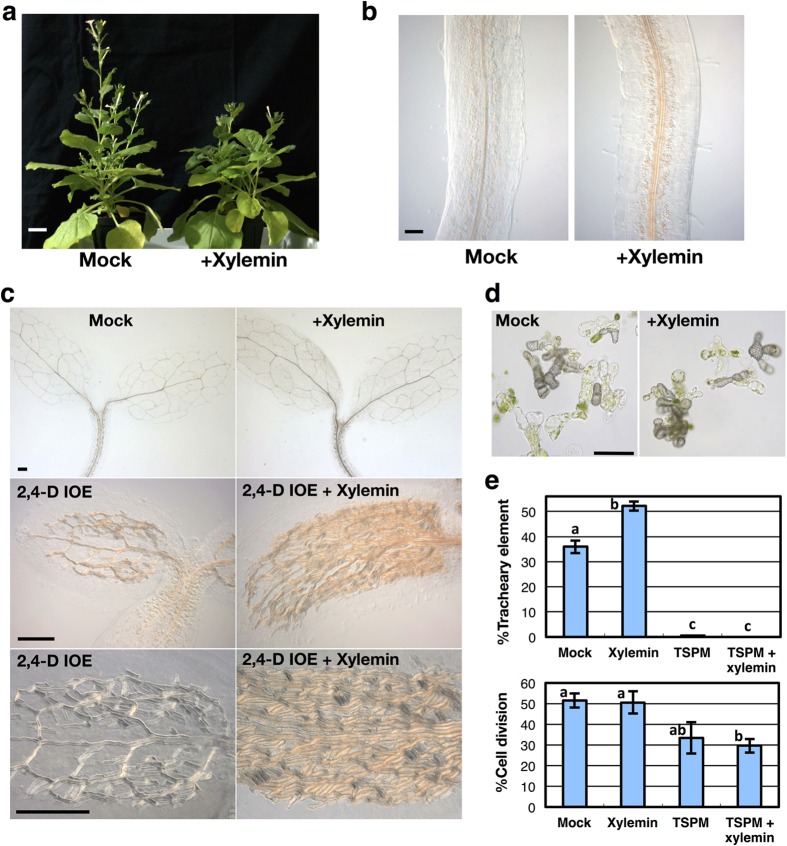
Xylemin remarkably promotes xylem differentiation in tobacco and zinnia. (**a**) Effect of xylemin on stem elongation of *N. benthamiana*. Xylemin solution at the concentration of 100 μM was daily applied to the shoot apex of *N. benthamiana*. Scale bar: 3 cm. (**b**) Effect of xylemin on xylem differentiation in hypocotyls of *N. benthamiana*. Seedlings of *N. benthamiana* were germinated and grown for 7 days in the liquid medium without (Mock) or with 50 μM xylemin (+Xylemin). Scale bar: 200 μm. (**c**) Effect of xylemin and 2,4-D IOE on xylem differentiation in cotyledons of *N. benthamiana*. Seedlings of *N. benthamiana* were germinated and grown for 7 days in the liquid medium without (Mock) or with 50 μM xylemin and/or 3 μM 2,4-D IOE. Scale bars: 200 μm. (**d**) Effect of xylemin on xylem differentiation in zinnia xylogenic culture. Isolated zinnia mesophyll cells were cultured in the differentiation medium without (Mock) or with 3 μM xylemin (+Xylemin) for 4 days. Scale bar: 100 μm. (**e**) Quantification of tracheary element differentiation and cell division in zinnia xylogenic culture. Isolated zinnia mesophyll cells were cultured in the differentiation medium without (Mock) or with 3 μM xylemin and/or 10 μM thermospermine (Tspm) for 4 days. Data are displayed as averages ± SD (*n* = 3). Different letters indicate Tukey’s HSD groupings (*P* < 0.05).

To test whether xylemin can be used for elucidating the functions of thermospermine and for inducing excessive xylem formation in other plants, we examined the effect of co-treatment with xylemin and 2,4-D IOE on the stem growth and xylem differentiation in *Nicotiana benthamiana*. Exogenously supplied xylemin remarkably suppressed stem elongation but enhanced xylem differentiation in *N. benthamiana* (Fig. 4a,b). Interestingly, simultaneous addition of xylemin and 2,4-D IOE strongly and ectopically induced xylem differentiation. This effect was especially remarkable in the cotyledons. Most mesophyll cells appeared to differentiate into xylem vessel cells, by which almost the entire cotyledon was covered (Fig. 4c).

Finally, we examined the effect of xylemin on xylogenic culture of *Zinnia elegans*. Isolated mesophyll cells of *Z. elegans* transdifferentiate into tracheary elements in the presence of auxin and cytokinin^29^. Xylemin treatment promoted tracheary element differentiation of *Z. elegans*, whereas thermospermine almost completely blocked it (Fig. 4d,e). Thermospermine also repressed cell division in the zinnia xylogenic culture (Fig. 4e), which is consistent with its negative effect on callus and lateral root formation (Fig. 2c). Therefore, thermospermine might negatively regulate cell division in the procambium/cambium and pericycle. These results support the notion that thermospermine-mediated suppression of auxin-inducible xylem differentiation is a universal mechanism for the regulation of xylem development in plants (Supplementary Fig. S6).

## Discussion

In this study, we developed xylemin as an inhibitor of thermospermine biosynthesis and showed that it can act as a strong inducer of xylem differentiation. Although xylemin might also slightly affect spermine biosynthesis and *SPMS* expression, we consider that this minor effect may not contribute to the manifested phenotype because spermine-deficient mutants of *Arabidopsis* show normal morphology and viability^16^. Expanding our previous finding that 2,4-D IOE enhances xylem differentiation in *acl5-1*, we found here that concomitant treatment with xylemin and 2,4-D IOE has a potent and synergistic inductive effect on xylem differentiation at least in dicotyledonous plants. The findings of this study further suggest that lateral root development and procambium/cambium formation also involve a feedback control mechanism in which auxin and thermospermine play opposing roles. Together with previous findings, we propose a model of the action of xylemin in auxin-triggered xylem differentiation (Supplementary Fig. S6). Auxin promotes xylem differentiation via the *MP*-*ATHB8* pathway^23^ and ATHB8 promotes *ACL5* expression^20^, resulting in the accumulation of thermospermine, which activates *SAC51* translation. Recent studies have shown that SAC51 interacts with a bHLH transcription factor, LONESOME HIGHWAY (LHW)^30^, whereas LHW interacts with another bHLH transcription factor TARGET OF MP5 (TMO5) to promote both vascular differentiation and *ACL5* expression^31^,^32^, suggesting an antagonistic effect of SAC51 on LHW-TMO5 heterodimerization. Because LHW is required for correct expression patterns of *MP* and *ATHB8*^33^, the LHW-SAC51 heterodimer might also directly or indirectly regulate these expression patterns in a feedback manner. Thereby, xylemin represses SAC51 translation by inhibiting thermospermine synthesis.

In the auxin canalization process, auxin accumulation enhances auxin transport, which in turn facilitates auxin accumulation into the cell^34^,^35^. This positive feedback loop generates directional and active flows of auxin molecules that determine the spatial pattern of vascular differentiation along the paths of auxin flow. Although the auxin canalization theory has been widely accepted as the primary mechanism for vascular pattern formation, it requires a pre-existing auxin source and some additional conditions or factors to form the closed vascular loops commonly found in leaf veins^34^,^35^,^36^. Thermospermine may be one of these factors. Because vascular tissues, especially xylem tissues, are enlarged in *acl5-1* and xylemin-treated wild-type plants (Figs 1 and 3), thermospermine function appears to narrow the auxin flow by generating an inhibitory field around it and limiting the zone of vascular cell differentiation. The dwarf phenotype of *acl5-1* and the phenocopy observed in xylemin-treated wild-type plants also suggest that the balance between differentiation of xylem vessel elements, which are eventually non-growing dead cells, and that of parenchymal tissues, is critical for organ elongation. Finally, our findings suggest that thermospermine is a major suppressor of xylem differentiation, which almost completely suppresses xylem formation in various organs and tissues. This is in contrast to the local effect of cytokinin on protoxylem specification in roots^37^,^38^ and the modest effect of the peptide hormone Tracheary element Differentiation Inhibitory Factor (TDIF) on xylem differentiation in roots and leaf veins^39^,^40^. Functional relationships between thermospermine and these local factors remain to be elucidated.

Manipulation of xylem differentiation is an important aspect of woody biomass production. Our findings suggest a significant possibility that xylemin and 2,4-D IOE can be applied to control woody biomass production without transgenic technology. Because thermospermine is present not only in vascular plants but also in nonvascular plants and some bacteria, xylemin is also expected to provide a useful tool for addressing physiological functions of thermospermine in these organisms.

## Methods

### Chemicals

Thermospermine was provided by Prof. Masaru Niitsu (Josai University). 2,4-D IOE was synthesized according to ref. 41 and provided by Prof. Kenichiro Hayashi (Okayama University of Science). 2,4-D IOE was dissolved in dimethyl sulfoxide (DMSO) and polyamines were dissolved in the sterilized water. They were adjusted so that the final concentration of DMSO in each medium was less than 0.1%. All other chemicals used in this study were purchased from Sigma-Aldrich (http://www.sigmaaldrich.com).

### Synthesis of xylemin

Xylemin (**1**, *N*-propylated putrescine) was synthesized by a four-step sequence from *N*-(2-Ns)-1,4-diaminobutane (**2**, Tokyo Chemical Industry, http://www.tcichemicals.com/en/jp/index.html) as a starting material (Supplementary Fig. S7). Thus, treatment of **2** with Boc_2_O/Et_3_N gave carbamate **3** in 98% yield. Selective alkylation of **3** was carried out with 1-bromopropane in the presence of Cs_2_CO_3_/TBAI to provide the desired propylated product **4**. The Ns group of **4** was removed with PhSH/Cs_2_CO_3_ to afford amine **5**. Finally, deprotection of the Boc moiety of **5** with SOCl_2_ proceeded smoothly to produce the desired xylemin (**1**). The purity (>95%) of the synthesized xylemin (**1**) was confirmed by its ^1^H NMR spectrum (Supplementary Fig. S8). The ^1^H NMR data of xylemin (**1**) were shown as followings: ^1^H NMR (400 MHz, D_2_O) *δ* 3.10–3.00 (m, 6 H), 1.77–1.65 (m, 6 H), 0.98 (t, *J* = 7.4 Hz, 3 H).

### Plant materials and growth condition

The *acl5-1* and *sac51-d* mutants were previously described^14^,^27^. *ATHB8:GUS* line was described in ref. 42 and provided by the Arabidopsis Biological Resource Center (ABRC). *ZCP4:GUS* line was described in ref. 43 and provided by Prof. Hiroo Fukuda (Tokyo University). Plants were grown at 22 °C in the modified Murashige-Skoog (MS) medium supplemented with 1% sucrose under continuous white light as described^17^,^44^. The liquid MS medium was used unless otherwise described. For the treatment with polyamines and/or 2,4-D IOE, 500 μl of MS liquid medium supplemented with polyamines and/or 2,4-D IOE was prepared in 24-well plates, and five to ten surface-sterilized *Arabidopsis* seeds (Columbia-0 accession) were spotted in each well, grown at 22 °C, and observed at the 7th day after germination for xylem vessel differentiation. Same liquid culture method was applied to *N. benthamiana*. For the experiment using MS agar medium, wild type *Arabidopsis* seeds (Columbia-0 accession) were spotted and grown on the MS medium that is supplemented with xylemin and/or thermospermine and solidified with 1% agar. For daily application of xylemin, 10–50 μL of 100 μM xylemin was added on the shoot apex of wild type *Arabidopsis* (Landsberg *erecta* accession) and *N. benthamiana*. Zinnia xylogenic culture was conducted according to ref. 29. The frequencies of tracheary element differentiation and cell division were calculated as the proportions of tracheary elements and divided cells to the number of living cells plus tracheary elements, respectively. The number of cells was counted in three biological replicates under a microscope (at least 500 cells were counted for each sample).

### Histology and microscopy

Seedlings were fixed in a 9:1 mixture of ethanol and acetic acid. Fixed samples were then cleared in a mixture of chloral hydrate, glycerol, and water solution (8 g : 1 ml : 2 ml) and observed under a differential interference contrast microscope (DM5000B, Leica, http://www.leica-microsystems.com) equipped with a CCD camera (DFC500, Leica). Root tissues were observed under a stereomicroscope (S8APO, Leica) equipped with a CCD camera (EC3, Leica). The seedlings of *ATHB8:GUS* and *ZCP4:GUS* were permeabilized by 90% acetone. Fixed samples were then incubated with a staining solution (0.5 mg/ml 5-bromo-4-chloro-3indolyl-β-Gluc, 5 mM K_3_[Fe(CN)_6_], 5 mM K_4_[Fe(CN)_6_], 0.1% Triton X-100, 50 mM sodium phosphate buffer (pH 7.2)) for 12 h and observed under the above mentioned light microscope or stereomicroscope. Cotyledons, hypocotyls, and GUS-stained seedlings were also observed under a light microscope (SMZ-ZT-1, Nikon, http://www.nikon-instruments.jp/jpn/index.html) equipped with a CCD camera (DS-L1, Nikon). Samples for cross section observation were fixed in FAA (45% ethanol, 5% formaldehyde, 5% acetic acid), dehydrated through an ethanol series, and embedded in Technovit 7100 resin (Heraeus Kulzer, www.heraeus-kulzer.com/). Samples were sectioned into 10-μm-thick slices by a rotary microtome (RM2245, Leica) equipped with a tungsten carbide disposable blade (TC65, Leica). Sections were stained with 0.1% Toluidine blue and observed under the light microscope (DM5000B). Inflorescence stems from one-month-old plants were embedded in 4% agar, sectioned by a vibrating blade microtome (MicroSlicer ZERO-1, D.S.K, http://www.dosaka-em.jp/), stained with phloroglucinol-HCl (1% phloroglucinol in 6 M HCl) and observed under the light microscope (DM5000B).

### Reverse transcription-quantitative polymerase chain reaction (RT-qPCR)

Total RNA was isolated from whole seedlings grown in the liquid medium for 7 days by phenol/chloroform extraction and subsequent lithium chloride precipitation as described^45^. For each sample, 30 ng of total RNA was reverse transcribed to cDNA using ReverTra Ace reverse transcriptase (TOYOBO, http://www.toyobo.co.jp) according to the accompanying protocol. Real-time PCR was performed in a DNA Engine Opticon2 System (Bio-Rad, http://www3.bio-rad.com) using KAPA SYBR FAST qPCR Kit (KAPA Biosystems, https://www.kapabiosystems.com) and gene-specific primers; 5′-ACCGT TAACC AGCGA TGCTT T-3′ and 5′-CCGTT AACTC TCTCT TTGAT TCTTC GATCC-3′ for *ACL5*, 5′-ATGGC AGTGT CTGGG TTCGA-3′ and 5′-CTATT TCCGA CGAGG CGTGA-3′ for *SAMDC4*, 5′-ACATA TCCAA GCGGC GTGAT-3′ and 5′-CCTCT TCAAG AGTTC TACAA AG-3′ for *SPMS*, 5′-AGCGT TTCAG CTAGC TTTTG AG-3′ and 5′-CAGTT GAGGA ACATG AAGCA GA-3′ for *ATHB8*, 5′-GATGA TCCAT GGGAA GAGTT-3′ and 5′-TAAGA TCGTT AATGC CTGCG-3′ for *MP*, 5′-ATGTG GCTAA AGGGA GTGAA-3′ and 5′-AACTT GCCAA ATCGA AACCC-3′ for *YUC2*, and 5′-GTGAG CCAGA TCTTC ATTCG TC-3′ and 5′-TCTCT TGCTC GTAGT CGACA G-3′ for *ACTIN8* (*ACT8*), according to ref. 19. Transcript levels of *ACT8* were used as a reference for normalization. The RT-qPCR were performed using at least three biological replicates.

### HPLC analysis

Polyamines were extracted from seedlings in 5% perchloric acid. Polyamine standards and plant samples were benzoylated according to ref. 46. The resulting samples were injected into a reverse-phase column (TSK-gel ODS-100V, 5 μm, 2.0 × 150 mm, Tosoh, Tokyo, Japan) and eluted with 42% (v/v) acetonitrile at a flow-rate of 0.2 mL/min for 30 min using the Agilent 1120 Compact LC. The benzoyl polyamines were detected at 254 nm. HPLC analyses were conducted in three biological replicates.

## Additional Information

**How to cite this article**: Yoshimoto, K. *et al.* Chemical control of xylem differentiation by thermospermine, xylemin, and auxin. *Sci. Rep.* **6**, 21487; doi: 10.1038/srep21487 (2016).

## Supplementary Material

Supplementary Figures
