# Supplementary material for: Chemical control of xylem differentiation by thermospermine, xylemin, and auxin
Source: Sci Rep. 2016 Feb 16;6:21487. doi: 10.1038/srep21487 (PMC4754900; doi:10.1038/srep21487)
Supplement: Supplementary Figures [file srep21487-s1.pdf]

Yoshimoto, K., Takamura, H., Kadota, I., Motose, H. & Takahashi, T.

Chemical control of xylem differentiation by thermospermine, xylemin, and auxin

Supplementary Figures S1-S8

Supplementary Fig. S1

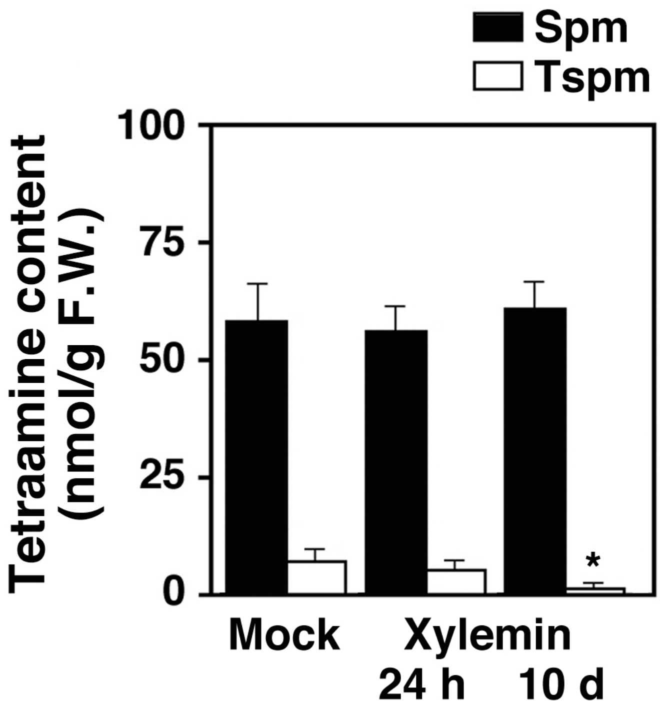

Supplementary Figure S1 | Tetraamine content in wild-type seedlings grown with xylemin. Wild-type seedlings were grown for 10 days in the MS liquid medium without xylemin (Mock), grown for 9 days and treated for 24 h with 30 µM xylemin (24 h), or grown for 10 days with 30 µM xylemin (10 d). Benzoylated polyamines were measured by HPLC. Data are displayed as averages ± SD (n = 3). Asterisk indicates a significant difference from the value in the mock treatment (Student *t*-test, $P < 0.01$).

Yoshimoto, K., Takamura, H., Kadota, I., Motose, H. & Takahashi, T.
Chemical control of xylem differentiation by thermospermine, xylemin, and auxin

Supplementary Fig. S2

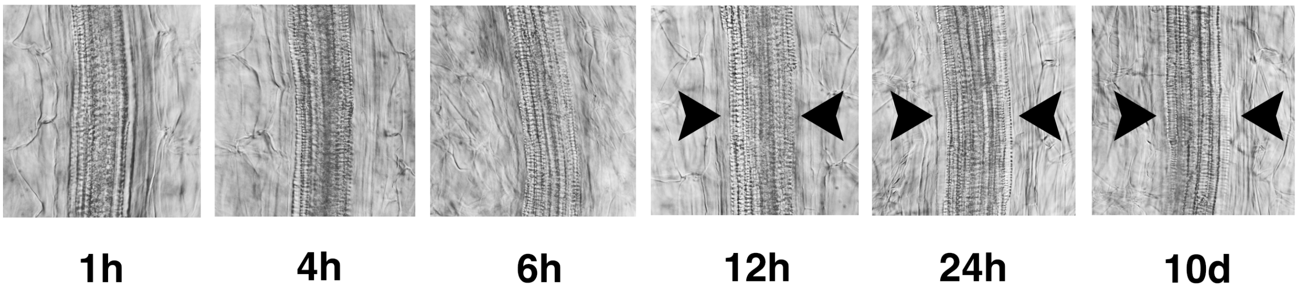

Supplementary Figure S2 | Changes in the responsiveness to xylemin during vascular development. The wild type seedlings were germinated and grown for 10 days in the liquid medium. Xylemin was added at various times of culture [1, 4, 6, 12, or 24 hours (h), or 10 days (at the start of culture) before fixation and observation] and xylem differentiation in hypocotyls was observed.

Yoshimoto, K., Takamura, H., Kadota, I., Motose, H. & Takahashi, T.
Chemical control of xylem differentiation by thermospermine, xylemin, and auxin

Supplementary Fig. S3

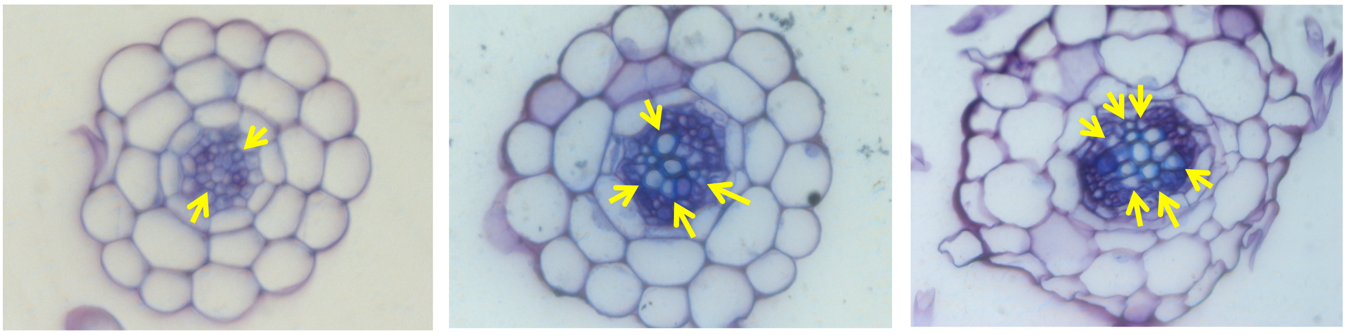

WT WT+xylemin *acl5*

Supplementary Figure S3 | Effect of xylemin on xylem differentiation in the root. Seedlings of the wild type were germinated and grown for 7 days in the medium without (WT) or with 50 μM xylemin (+ xylemin). The *acl5-1* mutant seedlings were germinated and grown for 7 days in the medium without xylemin. Arrows indicate xylem vessel elements.

Yoshimoto, K., Takamura, H., Kadota, I., Motose, H. & Takahashi, T.
Chemical control of xylem differentiation by thermospermine, xylemin, and auxin

Supplementary Fig. S4

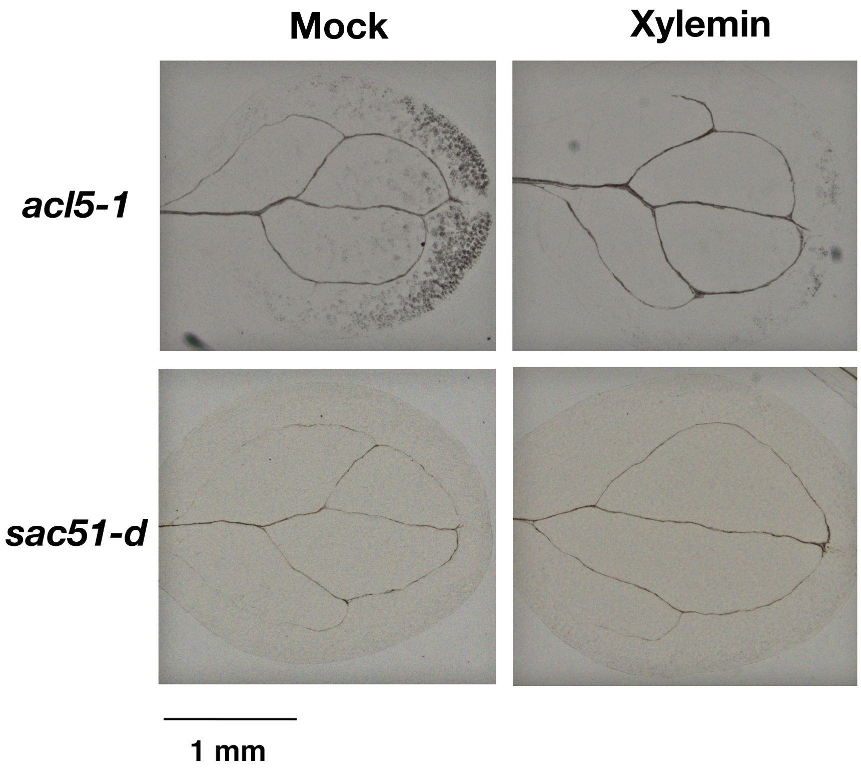

Supplementary Figure S4 | Effect of xylemin on xylem differentiation in *acl5-1* and *sac51-d*. Seedlings of the *acl5-1* or *sac51-d* mutants were germinated and grown for 7 days in the medium without (Mock) or with 50 µM xylemin (+Xylemin).

Yoshimoto, K., Takamura, H., Kadota, I., Motose, H. & Takahashi, T.
Chemical control of xylem differentiation by thermospermine, xylemin, and auxin

Supplementary Fig. S5

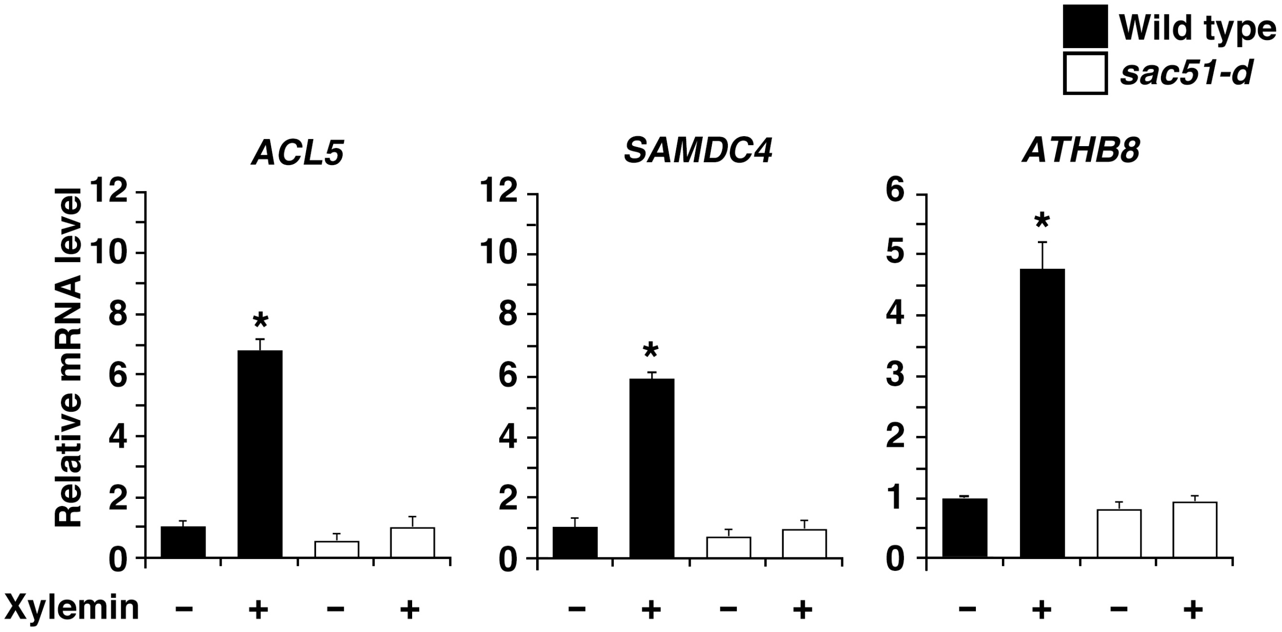

Supplementary Figure S5 | Effect of xylemin on gene expression in the wild type (WT) and *sac51-d*. Total RNA was isolated from seedlings of the wild type or *sac51-d* germinated and grown for 7 days in the liquid medium without (-) or with 50 µM xylemin (+). All transcript levels are relative to those in mock-treated seedlings. Data are displayed as averages ± SD (n = 3). Asterisk indicates significant difference from the value in the mock treatment (Student t-test, $P < 0.05$)

Yoshimoto, K., Takamura, H., Kadota, I., Motose, H. & Takahashi, T.
Chemical control of xylem differentiation by thermospermine, xylemin, and auxin

Supplementary Fig. S6

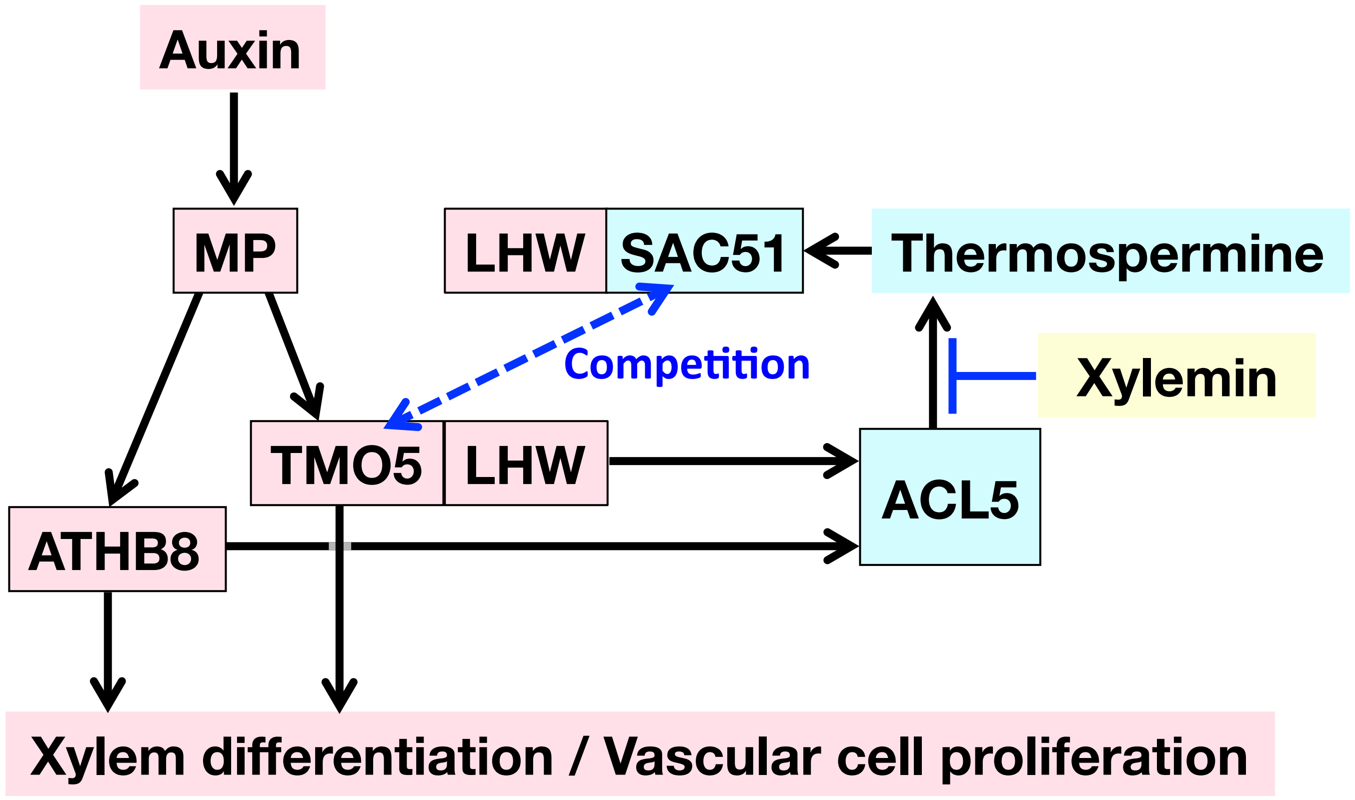

Supplementary Figure S6 | A model of thermospermine-mediated negative feedback regulation of auxin-inducible xylem differentiation. A putative model of thermospermine-mediated negative feedback regulation.

Yoshimoto, K., Takamura, H., Kadota, I., Motose, H. & Takahashi, T.
Chemical control of xylem differentiation by thermospermine, xylemin, and auxin

Supplementary Fig. S7

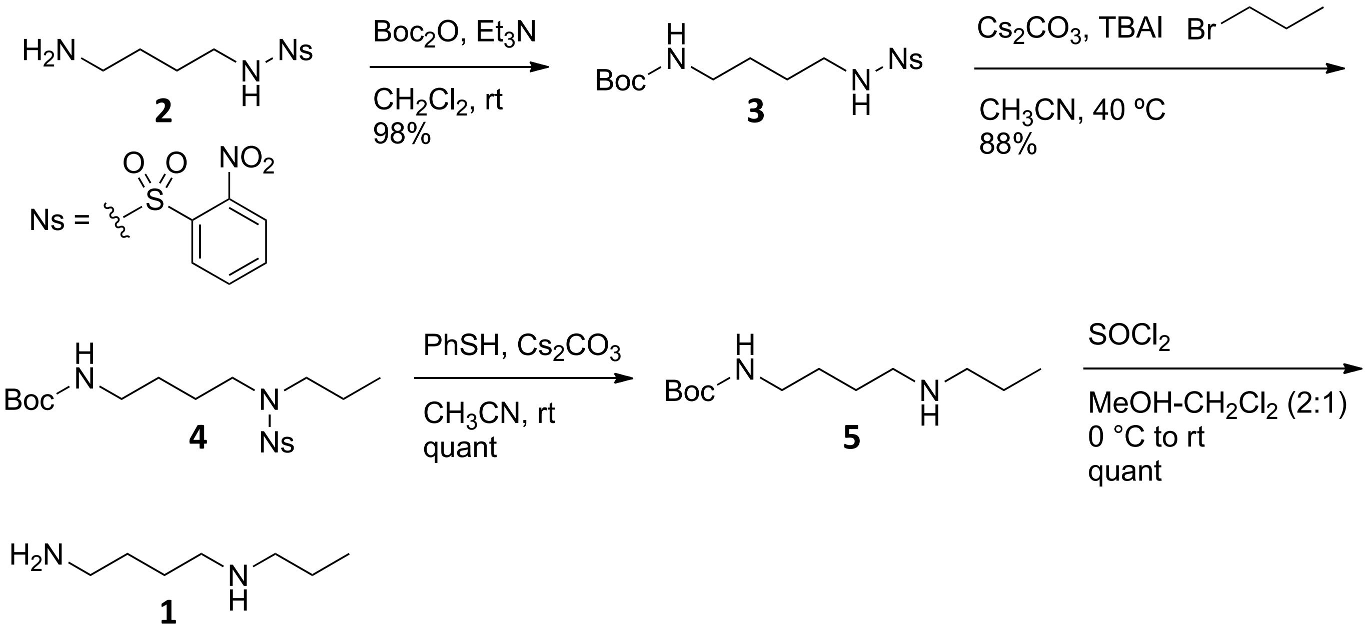

Supplementary Figure S7 | Synthetic scheme of xylemin.

Yoshimoto, K., Takamura, H., Kadota, I., Motose, H. & Takahashi, T.
Chemical control of xylem differentiation by thermospermine, xylemin, and auxin

Supplementary Fig. S8

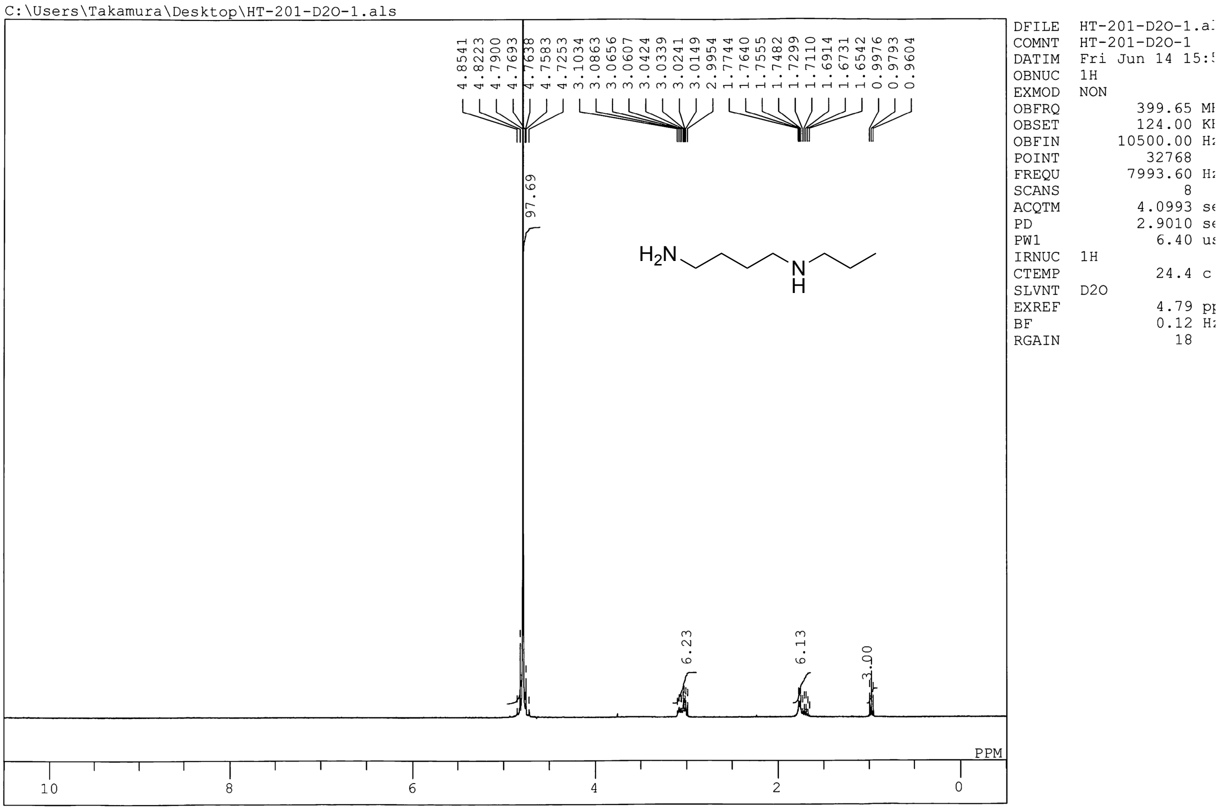

Supplementary Figure S8 | ^{1}H NMR spectrum of synthesized xylemin.

Yoshimoto, K., Takamura, H., Kadota, I., Motose, H. & Takahashi, T.
Chemical control of xylem differentiation by thermospermine, xylemin, and auxin
